# Rapid SNP Discovery and a RAD-Based High-Density Linkage Map in Jujube (*Ziziphus* Mill.)

**DOI:** 10.1371/journal.pone.0109850

**Published:** 2014-10-10

**Authors:** Jin Zhao, Jianbo Jian, Guannan Liu, Jiurui Wang, Minjuan Lin, Yao Ming, Zhiguo Liu, Yingying Chen, Xiuyun Liu, Mengjun Liu

**Affiliations:** 1 College of Life Science, Agricultural University of Hebei, Baoding, China; 2 BGI-Shenzhen, Shenzhen, China; 3 College of Life Science, Nankai University, Tianjin, China; 4 College of Forestry, Agricultural University of Hebei, Baoding, China; 5 Research Center of Chinese Jujube, Agricultural University of Hebei, Baoding, China; 6 National Agricultural Engineering Center for North Mountain Region of the Ministry of Science and Technology, Baoding, China; USDA-ARS-SRRC, United States of America

## Abstract

**Background:**

*Ziziphus* Mill. (jujube), the most valued genus of Rhamnaceae, comprises of a number of economically and ecologically important species such as *Z. jujuba* Mill., *Z. acidojujuba* Cheng et Liu and *Z. mauritiana* Lam. Single nucleotide polymorphism (SNP) markers and a high-density genetic map are of great benefit to the improvement of the crop, mapping quantitative trait loci (QTL) and analyzing genome structure. However, such a high-density map is still absent in the genus *Ziziphus* and even the family Rhamnaceae. The recently developed restriction-site associated DNA (RAD) marker has been proven to be most powerful in genetic map construction. The objective of this study was to construct a high-density linkage map using the RAD tags generated by next generation sequencing.

**Results:**

An interspecific F1 population and their parents (*Z. jujuba* Mill. ‘JMS2’ × *Z. acidojujuba* Cheng et Liu ‘Xing 16’) were genotyped using a mapping-by-sequencing approach, to generate RAD-based SNP markers. A total of 42,784 putative high quality SNPs were identified between the parents and 2,872 high-quality RAD markers were grouped in genetic maps. Of the 2,872 RAD markers, 1,307 were linked to the female genetic map, 1,336 to the male map, and 2,748 to the integrated map spanning 913.87 centi-morgans (cM) with an average marker interval of 0.34 cM. The integrated map contained 12 linkage groups (LGs), consistent with the haploid chromosome number of the two parents.

**Conclusion:**

We first generated a high-density genetic linkage map with 2,748 RAD markers for jujube and a large number of SNPs were also developed. It provides a useful tool for both marker-assisted breeding and a variety of genome investigations in jujube, such as sequence assembly, gene localization, QTL detection and genome structure comparison.

## Introduction

*Ziziphus* Mill. (jujube) is the best known Rhamnaceae genus of economic, ecological and cultural importance. Chinese jujube (*Z. jujuba* Mill., *2n*  =  24) and sour jujube (*Z. acidojujuba* Cheng et Liu, *2n*  =  24) are the most dominated cultivated and wild species of *Ziziphus*, respectively [1]. Chinese jujube, domesticated about 7,000 years ago in China [2], is one of the longest-cultivated fruit trees in the world. In 2012, the total production of Chinese jujube reached 5.4 million tons [3] with a growing area estimated 2 million hectares in China. It is also one of the most popularly used herbal medicines in Asia. Now it has become one of the most important dry fruits of China and has been introduced to more than 47 countries [4]. Sour jujube is the direct ancestor of Chinese jujube [2], [5] and provides a wide range of useful traits for the improvement of Chinese jujube and other *Ziziphus* species.

There is a rising need for high-quality jujube fruits. However, it usually takes decades to produce an advanced high-performing cultivar with the required traits using conventional cross-breeding approach in perennial woody fruit trees including jujubes. Marker-assisted selection (MAS) has been proven to be a promising solution for quickening breeding of fruit trees, and a high-density genetic linkage map can facilitate molecular marker development for high throughput selection of superior traits [6]. To date, there are only two available papers on jujube genetic maps. Shen (2005) constructed a primary genetic map using an intra-specific F1 population of *Z. jujuba* ‘Dongzao’ × *Z. jujuba* ‘Linyilizao’ with 150 progeny, which comprises 14 LGs spanning 1237.4 cM with 333 amplified fragment length polymorphisms (AFLP) markers [7]. Using the same F1 population, Qi et al. developed an improved Chinese jujube genetic map of 15 LGs spanning 1309.4 cM with 388 AFLP markers and 35 random amplified polymorphic DNA (RAPD) markers, with an average distance between markers of 3.1 cM [8]. The existing maps suffer from lack of markers (<450) and all of the mapped markers have no sequence information. Consequently, a completed high-density genetic map covering sufficient markers with sequence information is needed to meet the increasing demand for cultivar improvement and genetic research of jujube.

Early constructed genetic maps of plants using conventional molecular markers (RAPD, AFLP and Simple Sequence Repeats or SSR) generally included only a few hundred markers. The emergence of SNP marker provides exciting opportunities to construct saturated maps. The recently developed approach combining next-generation sequencing (NGS) and RAD enables thousands of SNP markers to be genotyped rapidly at relatively low cost. With the new technology, high density genetic maps of a number of plants including barley [9], eggplant [10], grape [6], field pea [11], *Legume* [12] and *Brassica napus* [13] have been developed.

Here, we first report a high-density genetic map of jujube using an inter-specific F1 population of *Z. jujuba* ‘JMS2’ × *Z. acidojujuba* ‘Xing 16’ and detect a large number of SNPs. It will facilitate the molecular breeding and a variety of genome investigations including sequence assembly, genome comparison, functional gene localization, QTL detection as well as genetic basis determination for the unique traits in jujube.

## Results and Discussion

### RAD Paired-end Contig Assembly and SNP Discovery

A total of 107 samples, including one female parent, one male parent and 105 F1 progenies, were sequenced for RAD paired-end contig assembly and further SNP identification. In RAD paired-end data, the sequenced ends near the restriction site are called RAD tags and the other ends are 2nd ends. The RAD tags have a restriction site at the end and the 2nd ends reads are randomly sheared. RAD sequence would allow the second reads to be assembled on a local basis, one RAD site at a time.

Some software has been developed to identify SNPs and define putative haplotypes in populations by locally assembling RAD tags [14]–[16]. By using Rainbow 2.0 [16], the parents’ RAD data were assembled into 177,381 contigs with a mean length of 266 bp (N50  =  266 bp, Table 1), which suggests most of the SNPs can be used to develop SNP assays for subsequent genotyping. The total length of the contigs is about 46.8 Mb which is approximately 10% of the jujube genome size (∼444Mb from k-mer analysis, unpublished). Using SOAP 2.20[17], the parents' RAD data were mapped to the reference contigs. 42,784 high-quality SNPs between the parents with sequencing depth of 5–150× and base quality of ≥ 25 were detected. Our sequences are available at the NCBI Short Read Archive (http://www.ncbi.nlm.nih.gov/Traces/sra/), at accession SRA176449/SRP044771.

**Table 1:** RAD paired-end contig assembly of the parents.

Total length (bp)	Total number	N90 (bp)	N50 (bp)	Maximum length (bp)
46,829,308	177,381	228	266	840

**Figure 1:**
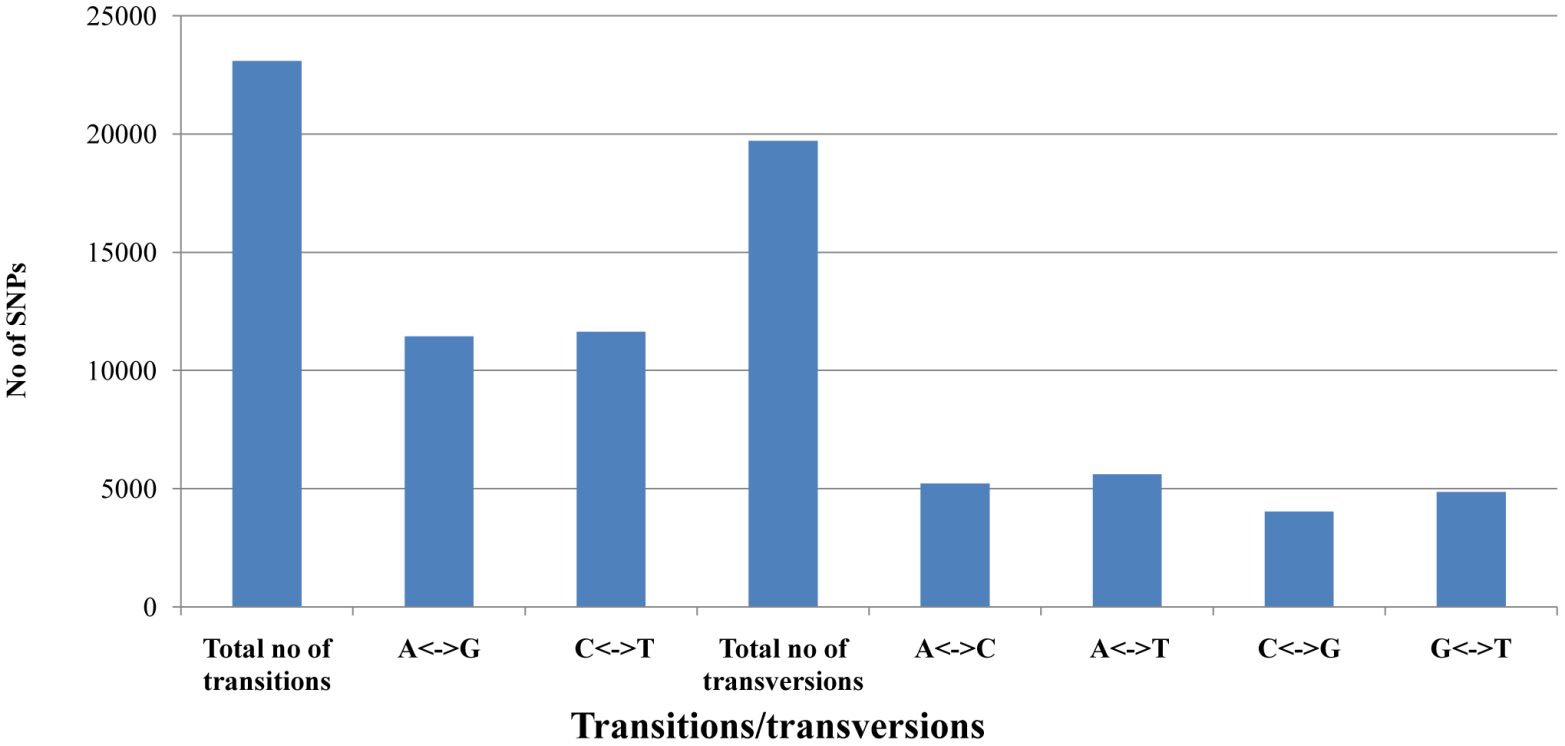
Transitions and transversions within 42,784 biallelic single nucleotide polymorphisms (SNPs) detected among jujube parents. This figure shows all the statistics of transitions and transversions SNPs between the jujube parents.

The nucleotide variation of 42, 784 SNPs in jujube were biallelic and consisted of 54% transitions and 46% transversions (Figure 1, Table S1), providing a ratio of 1.17, which is lower than that of grape (1.46) [18] and eggplant (1.65) [19], and higher than that of soybean (0.92) [20]. The number of different transitions was balanced (11,444 A/G and 11,638 C/T), and the number of transversions ranged from 4,030 (C/G) to 5,604 (A/T). The overall representation of different transitions (49.6% A/G and 50.4% C/T) and transversions (26.5% A/C, 28.4% A/T, 20.5% C/G and 24.6% G/T) through RAD sequencing we observed was in good accordance with those detected in eggplant (transitions: 49.7% A/G, 50.3% C/T, transversions: 24.0% A/C, 28.5% A/T, 19.9% C/G, 27.6% G/T) [19] and in *Brassica napus* (58.2% of transitions: 49.7% A/G and 50.3% C/T; 41.8% of transversions: 26.5% A/C, 29.7% A/T, 17.0% C/G and 26.8% G/T) [21]. Moreover, a total of 80 SNPs from the parents were validated by PCR and Sanger sequencing. Then, 76 SNPs were confirmed and 4 SNPs were erroneously inferred (Table S2). The correct rate for the SNP calling was about 95.0%.

### Stacks Analysis in the F1 Population

The Stacks software is an analysis tool set for population genomics [22], [23]. It was developed for the purpose of building genetic maps from RAD Tag Illumina sequence data and population studies. With the pipeline of Stacks version 0.9998, more than 3.1 million RAD tag sequences were generated in each of the two parents (Table S3). These sequences were clustered and counted resulting in more than 170,000 unique stacks in each parent (Table 2). This resulted in a set of 230,050 stacks and 135,855 shared stacks that contain both monomorphic and polymorphic RAD tags. 52,366 and 49,868 putative SNPs were found in female and male parents, respectively. 29,007 RAD markers between the parents were screened.

**Table 2:** RAD tag and SNP discovery in the parents.

Parent	Filtered RAD tags	Unique stacks	Shared stacks	Putative SNPs	Heterozygosity rate	RAD markers
**Female (P1)**	5,025,822	200,800	135,855	52,366	0.36%	29,007
**Male (P2)**	3,356,779	176,039	135,855	49,868	0.39%	29,007

Note: Filtered RAD Tags are the number of the reads with the specific recognition site (AATTC). The unique stacks are the number of clustered RAD tag sequences found in the data from each parent using ustacks with 2 mismatches. The shared stacks are the number of unique stacks shared between the two parents including both polymorphic and monomorphic loci. Putative SNPs are the stacks containing loci in each parent. Heterozygosity rate is calculated the ratio of the number of SNPs to the total length of the unique stacks in each parent. The RAD markers are the different loci between the two parents by matched the loci from each parent against the catalog with 10-fold minimum stack depth.

With respect to the RAD tags sequence, the heterozygosity rate in female parent *Z. jujuba* (∼0.36%) is a little lower than that of male parent *Z. acidojujuba* (∼0.39%). *Z. acidojujuba* is the wild relative species of *Z. jujuba* and has many useful traits for the improvement of *Z. jujuba*. The higher heterozygosity of *Z. acidojujuba* indicates more abundant genetic variation, which also supports that the wild-related species has higher genetic diversity.

**Figure 2:**
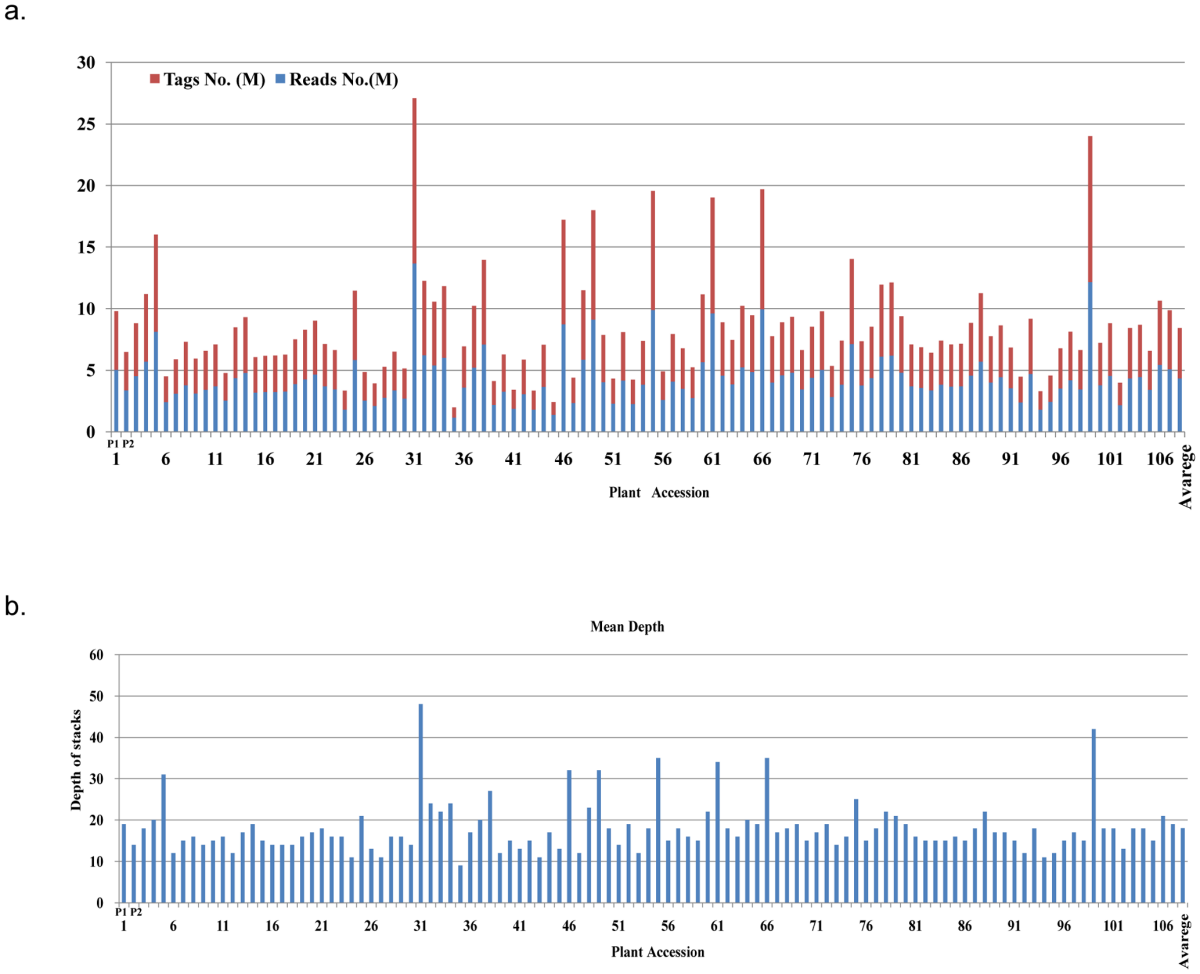
The statistics of reads, tags and stacks in 107 F1 plant accessions. (a) The number of tags and reads of the F1 population; (b) The mean depth of stacks in each individual.The ID 1 and 2 are the P1 (Female parent) and P2 (Male parent) respectively.

All the 107 F1 plant accessions (average 4.3 million reads per individual) have been clustered into stacks (Figure 2a) with minimum stack depth of 5. Finally, the depth of stacks in each sample is from 9 to 49, the average depth of stacks in all the samples is about 18 (Figure 2b). At the genotyping stage, a minimum stack depth of 10 reads was used to create a stack in an individual and a minimum stack depth of 15 to be called as homozygous.

### Construction of a Sequence-defined Genetic Map

**Figure 3:**
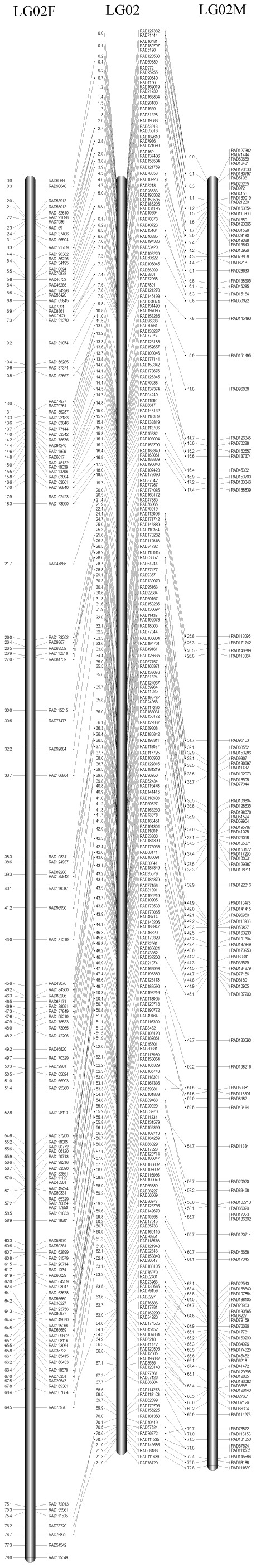
The linkage maps of LG02 for *Z. jujuba* (female, left), *Z. acidojujuba* (male, right) and their integrated map (middle) are shown as an example. Detailed lists of all the molecular markers, including their genetic distance in each linkage group, are presented in Table S4. All 12 LGs are presented in Figure S1, S2, and S3.

Only loci that were polymorphic within one or both parents could be mapped according to the double pseudo-test cross strategy [24], and the markers showing significantly distorted segregation (P-value <0.01) were excluded from the map construction. Linkage analysis was performed for markers present in at least 85% of individuals using JoinMap 4.0 with CP (cross pollination) population type codes [25]. At last, a total of 2,872 markers were screened out for map construction. Both the maps of female and the integrated map is comprise of 12 LGs which is consistent with the 12 chromosomes of jujube, the male parent map has 13 LGs because of LG04Ma and LG04Mb can not be grouped together, but the two groups can be aligned to the same group in the integrated map. Of the mapped markers, 1,307 fell into the 12 LGs for *Z. jujuba* (female), 1,336 for *Z. acidojujuba* (male), and 2,748 for the integrated map, with a grouping LOD (log of the odds) value of 5 to 10 (Figure 3 and Figure S1, S2, and S3, Table 3). The little difference in the number of markers between *Z. jujuba* (1,307) and *Z. acidojujuba* (1,336) is consistent with the heterozygosity rate in RAD tags (∼0.36% of *Z. jujuba* and ∼0.39% of *Z. acidojujuba*). To compare the order of the common markers, a homology plot-diagram (Figure S1, S2, and S3) was generated by lines with dots on both ends using the common marker on each parent map against the integrated map on the LGs. According to the analyses, most of the markers showed good linear agreement between the three maps on the basic framework. However, there were a small number of non-collinearity markers between the male, female and overall map. Therefore, the same order for the parent map most probably indicates conservation of genomes among the different jujube species; the non-consistent linear relationship for some LG regions might indicate some variations among different jujube species during evolution. This phenomenon also have been found in grape and *Brassica* [6], [26].

**Table 3:** A summary of the genetic linkage map constructed in *Ziziphus* Mill.

	Number of markers			Genetic sizes (cM)			Average marker interval (cM)		
	Integrated map	Female (P1)	Male (P2)	Integrated map	Female (P1)	Male (P2)	Integrated map	Female (P1)	Male (P2)
LG01	354	189	180	112.36	97.77	123.92	0.38	0.52	0.69
LG02	267	135	132	71.95	77.95	72.75	0.27	0.58	0.55
LG03	261	122	143	85.76	78.83	89.56	0.33	0.65	0.63
LG04	250	175	29	86.11	59.91	39.80	0.34	0.34	1.37
			11			16.11			1.46
LG05	237	94	101	69.26	73.29	56.68	0.29	0.78	0.56
LG06	234	86	122	65.54	63.66	62.46	0.28	0.74	0.51
LG07	219	87	108	89.92	81.79	85.90	0.41	0.94	0.80
LG08	207	84	110	65.45	62.18	70.01	0.32	0.74	0.64
LG09	204	82	124	71.95	76.34	73.02	0.35	0.93	0.59
LG10	195	94	102	73.40	74.64	76.40	0.38	0.79	0.75
LG11	167	60	104	66.49	72.52	67.62	0.40	1.21	0.65
LG12	153	99	70	55.68	44.50	84.68	0.36	0.45	1.21
**Total**	**2748**	**1307**	**1336**	**913.87**	**863.38**	**918.90**	**0.34**	**0.66**	**0.69**

Taking into account the size of all LGs, marker coverage amounted to 863 cM for *Z. jujuba* and 919 cM for *Z. acidojujuba* (Table 3). The average intervals between two adjacent mapped markers were 0.66 cM and 0.69 cM for the two parents, respectively. The integrated map spanned 913 cM, with average intervals between two adjacent mapped markers of 0.34 cM. The total physical size of the jujube genome was approximately 444 Mb (unpublished data), meaning that each 1,000-Kb DNA sequence was equal to an average of ∼2 cM genetic distance in this study. In other words, the average intervals between two adjacent markers were ∼340 Kb (444/1, 306 × 1, 000) for *Z. jujuba*, ∼332 Kb for *Z. acidojujuba* and 164 Kb for the integrated map.

Compared with the reported genetic map of Chinese jujube by Qi et al in 2009 (<450 markers, 15 LGs, an average interval of 3.1 cM between adjacent markers) [8], the integrated linkage maps we developed for the F1 population of *Z. jujuba* × *Z. acidojujuba* (2,748 markers, 12 LGs and an average interval of 0.34 cM) is of very high density. However, the total size of the previously reported Chinese jujube genetic map (1309 cM) that were mainly based on dominant markers using intra-species population of *Z. jujuba* is obviously larger than our map (913 cM), which is the opposite of the case in grape (more markers making a larger map size). There are several factors that can affect the genetic map length of a species, including map coverage, the mapping population, errors in genotyping and the choice of mapping functions.

Further analysis revealed that the markers on these 12 LGs were quite unevenly distributed. The maximum number of markers occurred on LG01, with 189 markers for the female, 180 for the male and 354 for the integrated map. The minimum number of markers occurred on LG11F for *Z. jujuba* (60), LG04Ma for *Z. acidojujuba* (11) and LG12 for the integrated map (153). The size of the LGs also varied widely (Table 3). The longest LGs were the same of LG01 for *Z. jujuba* (97.77 cM), *Z. acidojujuba* (123.92 cM) and the integrated map (112.36 cM). And the shortest LGs were LG12F (44.50 cM), LG04Ma (16.11 cM) and LG12 (55.68 cM) for *Z. jujuba, Z. acidojujuba* and the integrated maps, respectively. Adding more and varied markers for more individuals will further refine the map and improve its value for comparative mapping.

In addition, the 2,748 mapped markers combined with their 73-bp sequences (Table S4) could be used as shared anchors to compare genetic and physical maps, which would facilitate the application of jujube genomic resources.

## Conclusions

Using the next generation RAD sequencing approach we successfully generated the first high-density genetic map in both the genus *Ziziphus* Mill. and the family Rhamnaceae with an inter-specific F1 population of *Z. jujuba* Mill. ‘JMS2’ × *Z. acidojujuba* Cheng et Liu ‘Xing 16’. The integrated map spans 913.87 cM with 2,748 RAD markers distributed among 12 LGs, consistent with the haploid chromosome number expected for the species involved. It provides a tool for both molecular breeding and a variety of genome investigations in the genus, such as sequence assembly, gene localization, QTL detection and genome structure comparison.

## Materials and Methods

### Mapping Population and DNA Extraction

The F1 mapping population consists of 105 progenies from an inter-specific cross between two heterozygous genotypes, i.e., *Z. jujuba* Mill. ‘JMS2’ and *Z. acidojujuba* Cheng et Liu ‘Xing 16’. Since pollen abortion occurred in ‘JMS2’, ‘Xing 16’ was used as the male parent. The *in vitro* plantlets of the two parents and their progenies were preserved in the Research Center of Chinese Jujube, Agricultural University of Hebei, China. Leaf samples were harvested from F1 individuals and the two parents and immediately treated with liquid nitrogen and then stored at −80°C until DNA isolation. All the DNA samples were extracted using the DNeasy plant mini prep kit (Qiagen). The genomic DNA was treated with RNase to remove residual RNA.

### Library Construction and RAD Tag Sequencing

Genomic DNA (0.2–1.0 µg) was digested for 15 min at 37°C in a 50 mL reaction with 20 units (U) of *Eco*R I (New England Biolabs [NEB]). The used protocols of RAD tag sequencing were as described by Nathan A. Baird et al [27] except we used a modified Illumina P1 adapter containing individual specific nucleotide barcodes 4–8 bp long for sample tracking. All barcodes differed by at least two nucleotides to minimize sample miss-assignment due to sequencing error. Adapter-ligated fragments were subsequently pooled and randomly sheared (Bioruptor Branson sonicator 450) to an average size of 500 bp. Samples were then run out on a 1% agarose (Sigma), 0.5×TBE gel and DNA of 350 bp to 500 bp was isolated using a MinElute Gel Extraction Kit (Qiagen). After dsDNA ends were treated with end blunting enzymes and 3′-adenine overhangs were added, a modified Illumina P2 adapter was ligated. Finally, libraries were enriched by PCR amplification and RADs for each individual were sequenced on an Illumina Hiseq 2000 using paired-end reads (90bp). A sequencing depth of 10× for each RAD was desired in each sample. With the lack of jujube reference genome sequence, in-silico analysis of restriction enzyme-recognition sites can not be done, but with the *EcoR*I (GAATTC) can recognize 6 bases, if this restriction enzyme-recognition sites randomly distributed in this species, it will be about one recognition sites per 4Kb(4^6^). There may be 2.2 M reads can be enough for the average ten-fold depth of per RAD tag (444Mb/4Kb×2×10).

### RAD Sequence Data Analysis

Sequence reads from the Illumina were firstly quality-filtered by removing adapter pollutions in the reads and deletion of the reads containing more than 50% low quality bases (quality value ≤ 5). Then all reads were assigned to the individuals by the unambiguous barcodes and the specific recognition site (AATTC) with one mismatch. The reads without the unique barcodes and the specific sequence were discarded. Final 463.82 million clean reads were further trimmed to the RAD tags with uniform length of 82 nucleotides (nt). Each RAD tag comprised the 5nt of *Eco*R I recognition site and the 77 nt of potentially variable sequence. With the Rainbow 2.02 to assemble the parent RAD paired-end data (the length of first reads and second reads is 82 bp and 90 bp, respectively) [16]. The minimum number of reads used to generate a contig is 10. The contig below 200 bp was removed. SOAP2.20 [17] was used to map the parents' paired-end RAD reads onto the reference contig. SNPs were detected by SOAPsnp [28] to calculate the likelihood of genotypes of each individual. SNPs with a sequencing depth above 150 were removed as RAD sequences, which are too abundant, are likely to be repetitive sequences in the genome [14]. The retained SNPs are henceforth referred to as assay-compatible SNPs. The genotypes that were different between two parents were treated as potential high-quality SNP markers if the following criteria were satisfied: sequencing depth ≥5 and ≤50, base quality ≥25. With the Stacks version 0.9998 (ustack, a minimum stack depth of 5 was required to create a stack), RAD data showed a substantial increase in the total number of parents putative SNPs at the tails of the sequences (the last nine nucleotides from the positions 74 to 82) **(Figure S4)**, suggestive of sequencing errors as Pujolar, J. M described [29], which were consequently removed from the analyses. For subsequent analyses, final read length was trimmed to 73 nucleotides, and only the first (left) paired-read was used.

The DNA fragments created by RAD tag library preparation have a restriction site at one end and are randomly sheared at the other end, so the second paired-end reads are less suitable for calling SNPs because of a lower coverage than the first reads [14]. The clean reads were then used to assemble the RAD sequences into loci, alleles were identified and SNP was detected using the pipeline of Stacks version 0.9998 [22]. Firstly, the RAD tags were clustered into exactly-matching stacks. A minimum stack depth of 5 was required to create a stack respectively in parent and progenies, a maximum sequence mismatch of 2 was allowed between stacks to merge into a locus within an individual. Secondly, a catalogue was created of the parents' possible loci and alleles with a maximum sequence difference of 3 allowed. Thirdly, each individual was matched against the catalogue. Finally, in genotype stage, a minimum stack depth of 10 reads was used, which was the number of exactly matching reads that must be found to create a stack in an individual, automated corrections options was set as minimum stack depth of 15 to be called as homozygous, and correcting for the neglected heterozygote alleles due to their low coverage depth.

### SNP validation

A subset of 80 SNPs was selected for experimental validation by PCR and Sanger sequencing. The postion of SNP markers, their upstream and downstream sequences and primer pairs designed are presented in Table S2. PCR reactions contained 10 ng of genomic DNA in a 12.5 µl reaction with 5 µM of each primer pair. The amplification conditions were as follows: 94°C for 4 min, followed by 35 cycles of 94°C for 30 s, 55°C for 40 s and 72°C for 40 s, and a final elongation at 72°C for 10 min. PCR products were separated on 2.0% agarose gels in 1×TAE. Amplified fragments were cloned into pMD19-T vector and sequenced by GENEWIZ Company.

### Linkage Map Construction

The double pseudo-test cross strategy was applied [24]. Linkage analysis was performed for markers present in at least 85% of individuals using JoinMap 4.0 software with CP population type codes [25]. All the RAD markers have been loaded in the JoinMap 4.0, the ratio of marker segregation was calculated by Chi-square test. Markers showing significantly distorted segregation (P-value <0.01) were excluded from the map construction. All the 2, 872 RAD marker that can be marked as ef × eg, hk × hk, lm × ll and nn × np were used to generate integrated map. Two types of markers ef × eg and lm × ll could be mapped to female linkage maps, and ef × eg and nn × np could be mapped to male linkage maps. The female and male linkage maps were created by maternal and paternal population nodes. The 192 ef × eg markers were used for comparison between the integrated map and female or male map. To group all the 2,872 markers, logarithm of odds (LOD) score thresholds ≥ 5 were used. Unlinked markers and small linkage groups including less than 3 markers were excluded from further analysis. Linkage between markers, recombination rate, and map distances were calculated using the Kosambi mapping function and the regression mapping algorithm with a recombination frequency threshold of 0.5. The markers with suspect linkages were also excluded because they might be falsely grouped. At the end, 1 307, 1 336 and 2 748 markers were respectively identified as paternal, maternal and integrated, which enabled the construction of male-specific, female specific and integrated linkage maps.

## Supporting Information

Figure S1 **LG01-LG04 for the female (** ***Z. jujuba*** **), the male (** ***Z. acidojujuba*** **) and their integration linkage map.** This file shows the linkage group 01 to 04 and all the markers in vector graphics. The left of the bar is the genetic distance, the right of the bar is the name of each RAD marker. The common markers have been linked by lines and exhibit the relationship of the markers.
(TIF)

Figure S2 **LG05-LG09 for the female (** ***Z. jujuba*** **), the male (** ***Z. acidojujuba*** **) and their integration linkage map.** This file shows the linkage group 05 to 09 and all the markers in vector graphics. The left of the bar is the genetic distance, the right of the bar is the name of each RAD marker. The common markers have been linked by lines and exhibit the relationship of the markers.
(TIF)

Figure S3 **LG10-LG12 for the female (** ***Z. jujuba*** **), the male (** ***Z. acidojujuba*** **) and their integration linkage map.** This file shows the linkage group 10 to 12 and all the markers in vector graphics. The left of the bar is the genetic distance, the right of the bar is the name of each RAD marker. The common markers have been linked by lines and exhibit the relationship of the markers.
(TIF)

Figure S4 **The number of SNPs per nucleotide position (1–82).** There is an apparent increase in number of SNPs in the last nine nucleotides (74–82), suggesting of sequencing errors, which were consequently removed from the analyses.
(TIF)

File S1 **The contigs of the parents.** This text file lists in fasta format the assembled contigs (> =  200 bp) from the parents RAD paired-end reads of jujube.
(FA) Click here for additional data file.

Table S1 **The putative SNPs between two parents.** This file lists the polymorphisms called between the jujube parents, using the File S1 contig files as the reference. Explanation of output: Each SNP is described by one line of the table. It includes the name of contig; the postion of SNP in the contigs; the genotype, mapping value and coverage depth of each parent.
(XLS)

Table S2 **SNP validation using PCR and Sanger sequencing.** This table file contains the name of contig, the upstream and downstream sequences of SNPs, the postion of SNP loci, the genotype of each parent and the primers designed. 76 SNPs were verified by Sanger sequencing, and 4 SNPs marked by yellow background were erroneously inferred.
(XLS)

Table S3 **The data production, quality, alleles and tags of F1 population.** This table lists all the basic statics of all the 107 accessions including the sequenced data; the quality rate (Q20 and Q30); the read length deal with following the result of Figure S4 and alleles and tags which were obtained from basic analysis result by the software of stacks.
(XLSX)

Table S4 **The genetic distances and consensus sequence of RAD markers in the integration map of jujube.** This table lists all the genetic distances and markers of each linkage group. All the RAD marker in the final linkage group have been found in an unique consensus sequence, the haplotype of each parents in the tag and the type of marker also have been showed in the table.
(XLS)
