# Supplementary figures and images for: Rapid SNP Discovery and a RAD-Based High-Density Linkage Map in Jujube (Ziziphus Mill.)
Source: PLoS One. 2014 Oct 10;9(10):e109850. doi: 10.1371/journal.pone.0109850 (PMC4193841; doi:10.1371/journal.pone.0109850)

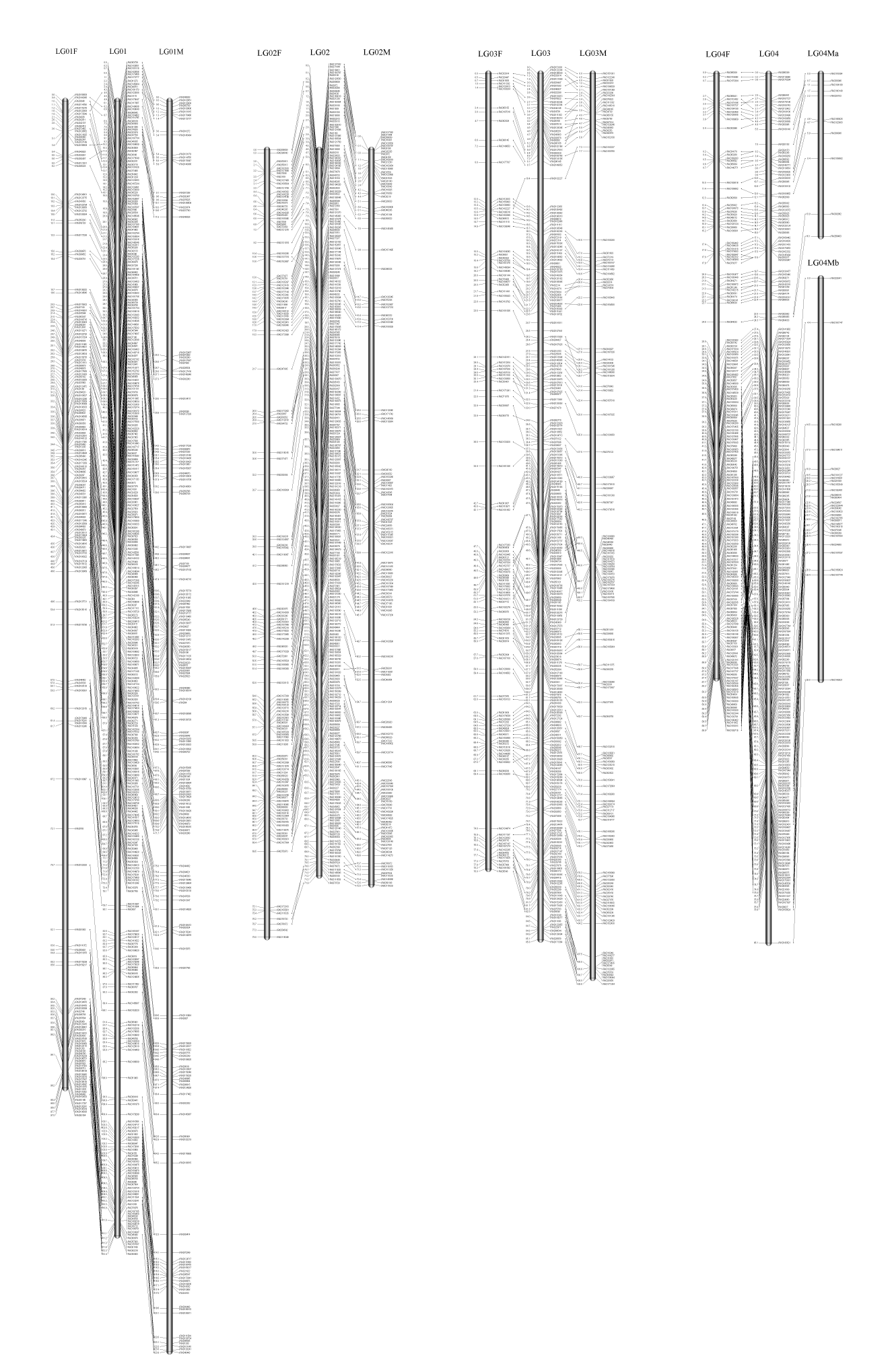

Supplement: Figure S1 — LG01-LG04 for the female ( Z. jujuba ), the male ( Z. acidojujuba ) and their integration linkage map. This file shows the linkage group 01 to 04 and all the markers in vector graphics. The left of the bar is the genetic distance, the right of the bar is the name of each RAD marker. The common markers have been linked by lines and exhibit the relationship of the markers. (TIF) [file pone.0109850.s001.tif]

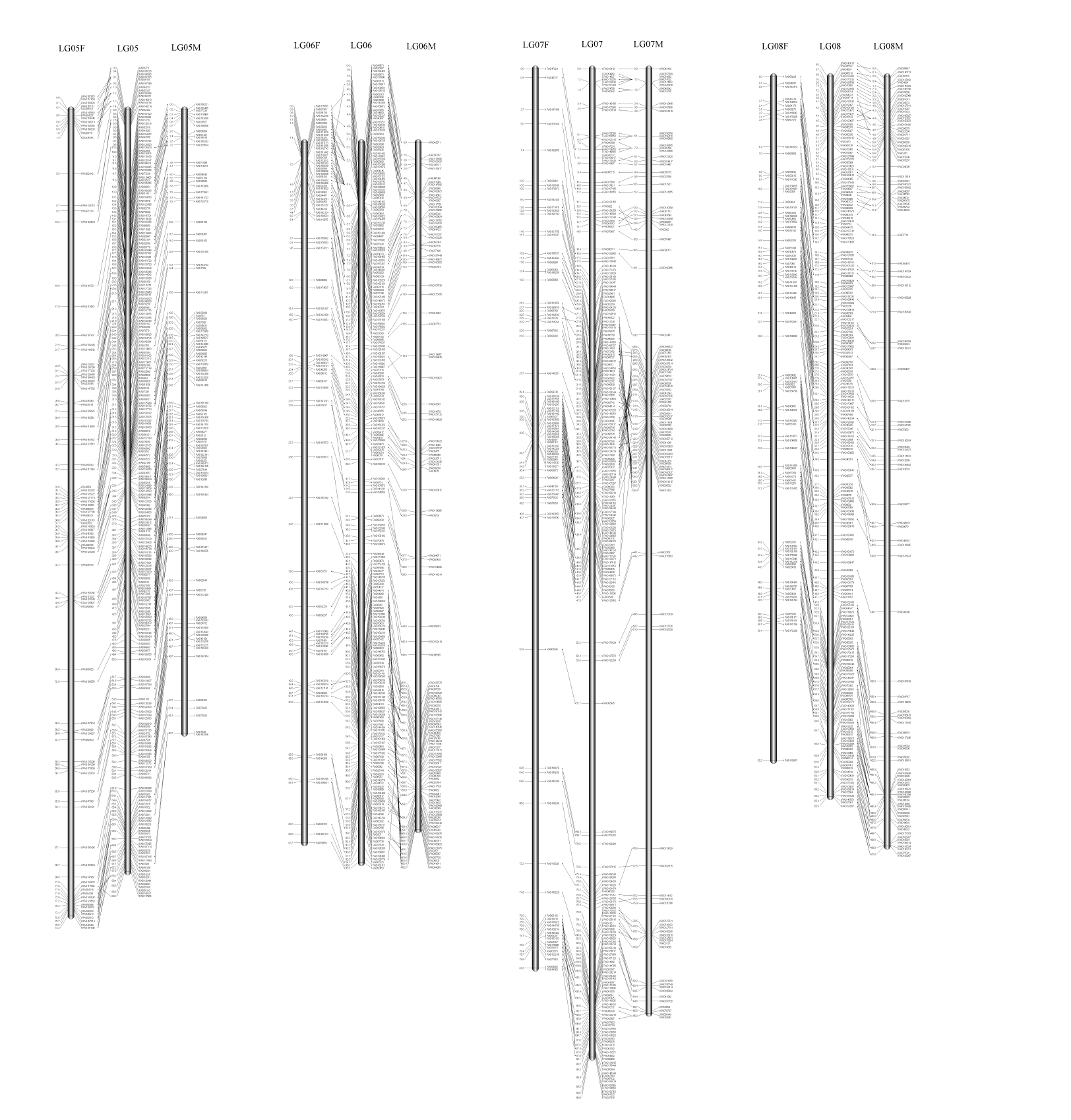

Supplement: Figure S2 — LG05-LG09 for the female ( Z. jujuba ), the male ( Z. acidojujuba ) and their integration linkage map. This file shows the linkage group 05 to 09 and all the markers in vector graphics. The left of the bar is the genetic distance, the right of the bar is the name of each RAD marker. The common markers have been linked by lines and exhibit the relationship of the markers. (TIF) [file pone.0109850.s002.tif]

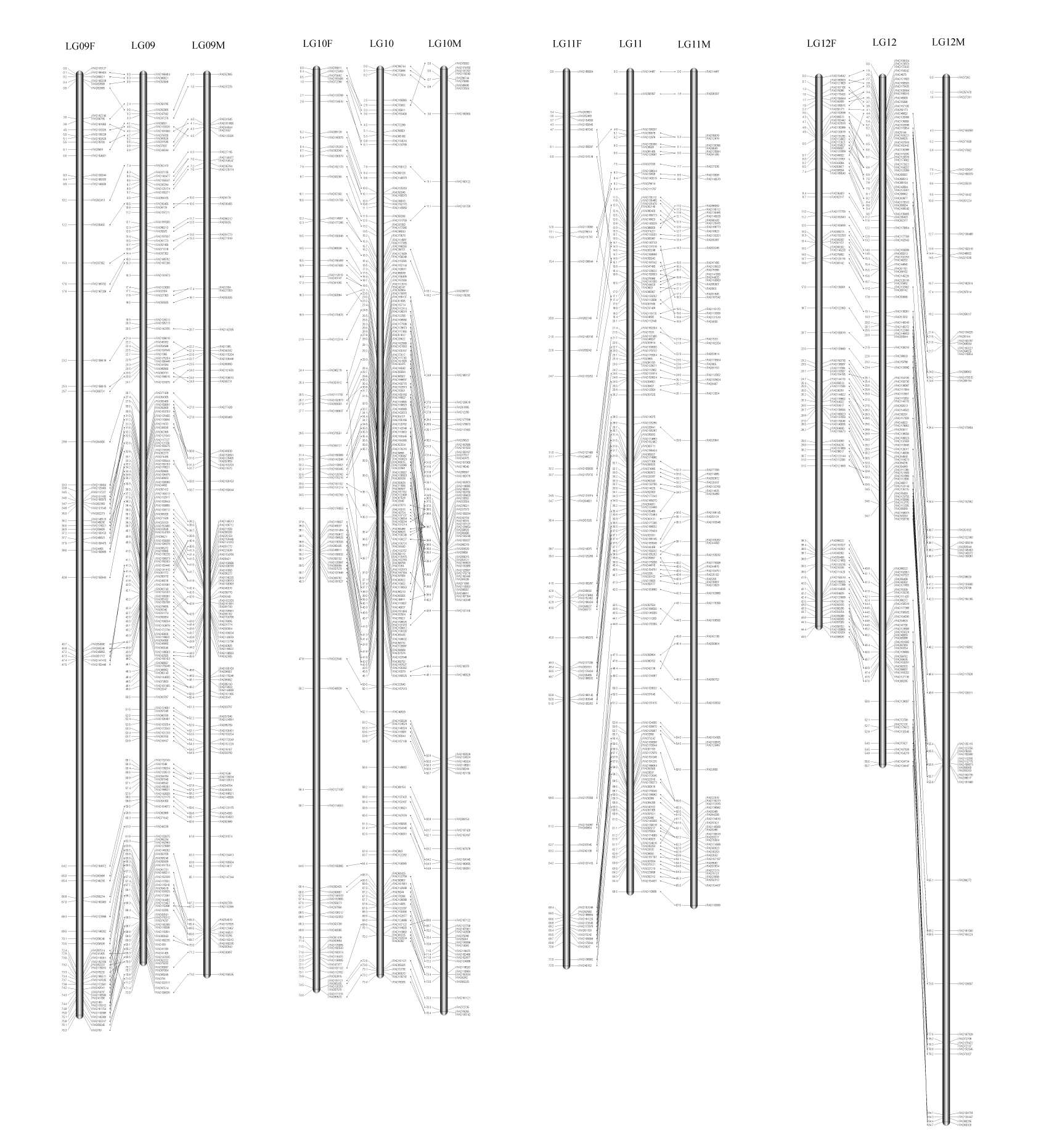

Supplement: Figure S3 — LG10-LG12 for the female ( Z. jujuba ), the male ( Z. acidojujuba ) and their integration linkage map. This file shows the linkage group 10 to 12 and all the markers in vector graphics. The left of the bar is the genetic distance, the right of the bar is the name of each RAD marker. The common markers have been linked by lines and exhibit the relationship of the markers. (TIF) [file pone.0109850.s003.tif]

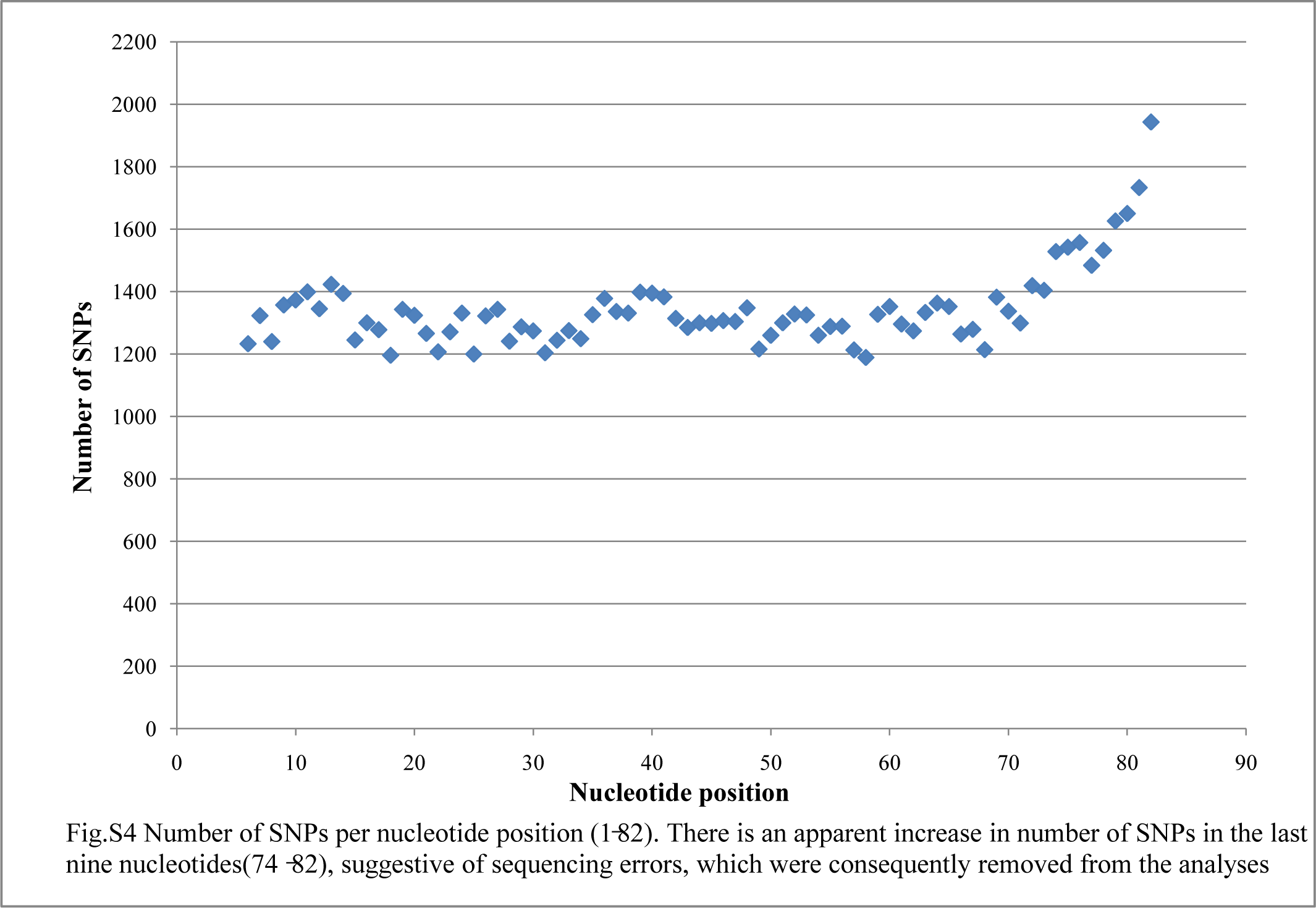

Supplement: Figure S4 — The number of SNPs per nucleotide position (1–82). There is an apparent increase in number of SNPs in the last nine nucleotides (74–82), suggesting of sequencing errors, which were consequently removed from the analyses. (TIF) [file pone.0109850.s004.tif]
